# The health needs and healthcare experiences of young people trafficked into the UK

**DOI:** 10.1016/j.chiabu.2016.08.001

**Published:** 2016-09

**Authors:** Nicky Stanley, Siân Oram, Sharon Jakobowitz, Joanne Westwood, Rohan Borschmann, Cathy Zimmerman, Louise M. Howard

**Affiliations:** a School of Social Work, Care and Community, University of Central Lancashire, Preston, PR1 2 HE, UK; b Institute of Psychiatry, Psychology & Neuroscience, King’s College London, London, SE5 8AF, UK; c School of Applied Social Science, University of Stirling, FK9 4LA, UK; d Murdoch Childrens Research Institute, Royal Children’s Hospital, Melbourne 3052, Australia and Melbourne School of Population and Global Health, University of Melbourne, Victoria 3010, Australia; e Department of Global Health and Development, London School of Hygiene and Tropical Medicine, Keppel Street, London WC1E 7HT, UK

**Keywords:** Trafficked, Young people, Health, Healthcare

## Abstract

Young people who have been trafficked may have experienced significant trauma and violence but little is known about their health and healthcare needs. This UK study aimed to address that gap. It included a health survey and qualitative interviews with 29 young people aged 16–21 trafficked into the UK from other countries who were recruited through voluntary organisations and children’s social services. These data were supplemented by interviews with relevant professionals. Over half the young people had been trafficked for sex work but sexual violence had also been experienced by those trafficked for domestic servitude and labour exploitation. Physical violence, threats, restrictions of liberty and deprivation were also widespread, as were experiences of physical and sexual violence prior to being trafficked. Five young women had become pregnant whilst trafficked; three were parents when interviewed. Two-thirds screened positive for high levels of psychological distress, including PTSD. Twelve reported suicidal thinking. Whilst some were keen for opportunities to talk to health professionals confidentially and wanted practitioners to treat their accounts as credible, others wanted to forget abusive experiences. Complex gatekeeping systems, language barriers and practitioners who failed to take them seriously limited access to healthcare. Support and advocacy were helpful in assisting these young people to navigate healthcare systems. Health professionals need to recognise and respond appropriately to trafficked young people’s often complex mental health needs and refer them to relevant services, as well as facilitating care at later times when they might need support or be more ready to receive help.

## 1 Introduction

Human trafficking is “the recruitment, transportation, transfer, harbouring or receipt of persons by means of threat or use of force or other forms of coercion, of abduction, of fraud, of deception, of the abuse of power, or of a position of vulnerability or of the giving or receiving of payments or benefits to achieve the consent of a person having control over another person, for the purpose of exploitation” (United Nations, 2000). Trafficking is believed to affect every country of the world, as countries of origin, transit or destination, and the International Labour Office (ILO) estimates that up to 20.9 million people worldwide may be in situations of forced labour as a result of human trafficking (ILO, 2012). In the UK, the Modern Slavery Act 2015 addresses both human trafficking and slavery, defining slavery as knowingly holding a person in slavery or servitude or knowingly requiring a person to perform forced or compulsory labour. An offence of human trafficking is committed if a person arranges or facilitates the travel of another person with a view to that person being exploited, where exploitation refers to slavery, servitude, forced or compulsory labour, sexual exploitation, removal of organs, or the securing of services by force, threats, deception or from children or vulnerable persons. The United Nations Palermo Protocol (United Nations, 2000), which includes the definition quoted above, established that children and young people under 18 cannot consent to their own exploitation regardless of the degree of coercion involved; this concept has been incorporated into UK guidance (DCSF, 2007).

The covert and illegal nature of trafficking, together with challenges in achieving a consistent definition, makes for difficulties in measuring its prevalence (CEOP, 2007, CEOP, 2009). Some indication of the scale of the issue in the UK can be obtained from figures provided by the UK National Referral Mechanism (NRM) which provides the route through which trafficked people can apply for temporary immigration protection, accommodation and support (Home Office, 2014). In 2014, 671 children and young people under 18 were referred into the NRM; the most common countries of origin were Albania, Vietnam, the UK, Slovakia, and Nigeria (NCA, 2015). However, this is far from a full picture since this only includes those who have exited from the trafficking situation and are in contact with statutory and voluntary agencies permitted to make referrals on behalf of children they suspect may have been trafficked. Fears of recriminations from traffickers and/or arrest or deportation by the authorities can act as barriers to help-seeking and use of official agencies (Pearce, 2011). Adolescents’ mistrust may be heightened further because they are often obliged to prove their status as children in order to access support from children’s social services. Many fear that they will lose their right to stay in the UK at the age of 18 (Crawley, 2007).

The experiences and needs of trafficked children and young people have also been difficult for researchers to capture and there are similar reasons for this, although high levels of vulnerability together with gatekeepers’ concerns about the safety and confidentiality of this group may also play a part. Few studies have been able to access trafficked young people directly (Ottisova, Hemmings, Howard, Zimmerman, & Oram, 2016). One exception is Kiss et al.’s (2015) survey of 387 10–17 year olds in the Greater Mekong Subregion. The authors found that the girls participating in their study had been trafficked primarily for forced sex work. Over half the young people (56%) in their sample reported symptoms indicative of depression, one in three had symptoms of an anxiety disorder and 12% had tried to harm or kill themselves in previous month. In the UK, Franklin and Doyle (2013) interviewed 17 young people aged between 15 and 23 who had been trafficked as children. They also surveyed local authorities and completed telephone interviews with key stakeholders. Their findings identified a high level of need for mental health services and highlighted poor continuity of care for trafficked children who had to retell their histories of abuse and exploitation to numerous social workers. Some evaluations of initiatives for young people who are either asylum seekers or trafficked, such as Crawley and Kohli’s (2013) largely positive evaluation of the Scottish Guardianship pilot service included interviews with and case file studies of small numbers of young people who had been trafficked. This study found that Guardians could play a key role in assisting these young people to navigate and access health services. Varma, Gillespie, McCracken, and Greenbaum’s (2015) US study used case file review to study 84 children aged 12–18 presenting at hospital emergency departments or at a child protection clinic, of whom 27 were defined as victims of commercial child sexual exploitation or trafficking. Over 50% of this group had had a sexually transmitted infection (STI) and they were more likely than a comparison group of sexually abused young people to have experienced violence and to have a history of drug use. A similarly high rate of STIs (35%) was found by Crawford and Kaufman (2008) who studied the case files of 20 sexually exploited adolescent females receiving post-trafficking NGO support in Nepal.

Other studies have focused on the knowledge and perceptions of practitioners working with trafficked children and young people with the aim of improving identification and service provision for this group. Pearce (2011) completed focus groups and interviews with 72 UK practitioners and analysed 37 case studies from the files of a child trafficking advice and information service. She identified a ‘wall of silence’ constructed from children’s anxieties associated with talking about their experiences and practitioners’ lack of knowledge of indicators of trafficking or their disbelief of children’s accounts. Together, these made for difficulties in identifying and responding to trafficked children and young people. She found that practitioners were sometimes unable to distinguish between smuggling and trafficking and that there was potential for the sexual exploitation of trafficked boys to be overlooked. Ross et al.’s (2015) survey of 782 health professionals in England found that over half (55%) did not feel confident that they could make appropriate referrals for trafficked children. Eighty per cent of the sample considered that they had not received sufficient training to be able to assist individuals whom they suspected might be trafficked. Cole and Sprang’s (2015) study identifies the uneven nature of the response to trafficked young people. They completed a telephone survey with 289 professionals in metropolitan, micropolitan and rural areas in the US. While they found practitioners across all areas reported similarities in the situations of children and young people who had been trafficked for sexual exploitation, professionals in metropolitan areas were more likely to have experience of working with victims of sex trafficking, to have received appropriate training, be familiar with relevant legislation and to perceive it as a fairly or very serious problem.

This mixed methods study was planned to provide an in-depth picture of the health needs and healthcare experiences of young people in England who had recently been trafficked from other countries. We also aimed to understand the challenges they faced in accessing health services through exploring both young people’s and professionals’ perceptions of the barriers and enablers to healthcare provision. The study was part of a larger programme of research which also included a survey of the health needs and experiences of trafficked adults (Oram et al., 2016) as well as a two systematic reviews (Hemmings et al., 2016; Ottisova et al., 2016), analysis of the characteristics of trafficked adults and children in contact with secondary mental health services (Oram, Khondoker, Abas, Broadbent, & Howard, 2015) and a survey of healthcare professionals’ knowledge and attitudes towards human trafficking (Ross et al., 2015).

## 2 Methods

### 2.1 Recruitment

We aimed to recruit trafficked young people aged 14–21 who were no longer in the setting where they had been exploited. Young people were identified through voluntary sector organisations providing post-trafficking support and children’s social services departments located in London and in the South, South East, West and North West of England. Support workers at participating organisations approached potentially eligible participants and provided them with basic information about the study; written information was available in multiple languages. Interviews were scheduled with assistance from support workers, with the verbal consent of potential participants. Written consent was obtained by researchers prior to conducting face-to-face interviews. Young people were interviewed either in their current accommodation or in agency premises. Professionally qualified, independent interpreters were used in nine of the 29 interviews and four young people chose to have their carer or support worker present during the interview. The length of interviews varied between 60 and 120 min depending on whether an interpreter was used, whether breaks were taken and the extent of the young person’s use of healthcare services. All participants were given a £20 high street shopping voucher to thank them for their participation and travel and childcare expenses were reimbursed. Attention has been given to protecting their confidentiality and anonymity and ethical approval for the study was provided by the National Research Ethics Service (reference 13/LO/0099).

Qualitative face to face interviews were carried out with health practitioners and professionals working outside the health sector, including civil servants, voluntary sector organisations, police officers, and members of the UK Human Trafficking Centre. These aimed to explore professional perceptions of the barriers and facilitators to trafficked people’s access to healthcare. Eligible professionals were identified with assistance from advisory groups and local collaborators and by snowballing (Noy, 2008). A purposive approach was taken to sampling with the aim of recruiting a variety of health professionals from relevant settings and a range of local and national stakeholders from welfare, legal and security services. Interviews were recorded and professionally transcribed. In this paper, we draw on data from seven of these interviews that addressed service provision for children and young people.

### 2.2 The health survey

The health survey was completed face-to-face which enabled the interviewer to provide explanations and reassurance when needed. Data were collected on socio-demographic factors, pre-trafficking and trafficking experiences including exploitation type, duration of exploitation, time since escape, living and working conditions and violence, with questions devised in line with other studies in this field (e.g. Kiss et al., 2015). Medical history (including psychiatric disorder) was assessed using questions from the 2007 English Adult Psychiatric Morbidity Survey (National Centre for Social Research and University of Leicester, 2011). Physical symptoms were assessed using the Miller Abuse Physical Symptoms and Injury Survey (Miller and Campbell, 1993). Severe symptoms were defined as symptoms which bothered the participant “quite a lot” or “extremely” (versus “not at all” or “a little”). Questions adapted from the third UK National Survey of Sexual Attitudes and Lifestyles (Natsal, 2015) were used to ask about sexual and reproductive health.

For participants aged 18 and above, probable depressive disorder was assessed as a score of 10 or more on the Patient Health Questionnaire-9 (PHQ-9; Kroenke, Spitzer, & Williams, 2001), and probable anxiety disorder as a score of 10 or more on the Generalized Anxiety Disorder 7 (GAD-7; Spitzer, Kroenke, Williams, & Lowe, 2006). For under-18s, psychological distress was assessed as scores above 5, 4, and 6 on the emotional difficulties, conduct difficulties, and hyperactivity subscales of the Strengths and Difficulties Questionnaire (SDQ) respectively, or probable PTSD. Participants were categorised as having high levels of psychological distress if they screened positive for one or more of probable depressive disorder, anxiety disorder or above threshold scores on the SDQ.

Probable PTSD was assessed for both over- and under-18 s as a score of 3 or more on the 4 item version of the PTSD Checklist-Civilian (PCL-C; Prins, Ouimette, & Kimerling, 2004). For all participants, suicidality was measured using the Revised Clinical Interview Schedule (CIS-R; Lewis, Pelosi, Araya, & Dunn, 1992): participants who endorsed two or more items on the suicidality sub-section were categorised as suicidal.

Completion of the health survey was immediately followed by a series of open questions exploring experiences of accessing and using health services. This part of the interview was audio-recorded with young people’s consent and professionally transcribed.

### 2.3 Interviews with professionals

A topic guide was developed with assistance from the study’s national advisory group, piloted with four participants and revised accordingly. Topics addressed included experiences of referring and assisting trafficked people to access healthcare; opportunities, barriers, feasibility and recommendations for coordination between the NHS and other aspects of the UK response to human trafficking; and relevant examples from their own practice. Interviews were conducted face to face, digitally recorded and transcribed verbatim.

### 2.4 Analysis

The software package STATA 11 was used to analyse the quantitative data. Descriptive statistics (proportions for categorical variables, and either means and standard deviations, or medians and inter-quartile ranges (IQR) for continuous variables) were used to describe socio-demographic and trafficking characteristics and other variables of interest. The qualitative data from interviews with young people and from the interviews with professionals on access and use of health services were stored and sorted with the assistance of NVivo, and a Framework approach was adopted for the analysis (Smith & Firth, 2011) with a coding frame that incorporated both questions from the interview schedule and emerging themes. Coding was discussed by research team members and the coding frames were refined and revised accordingly.

## 3 Findings

### 3.1 Trafficked young people’s characteristics

In the event, we were not successful in recruiting any young people aged under 16. As is often the case when researching sensitive issues with a vulnerable population of children or young people (Munro, Holmes, & Ward, 2005), recruitment was a demanding process as local authorities lacked the systems that would enable them to search their records for trafficked young people, gatekeepers were anxious about requiring young people to retell their story and some young people were reluctant to participate in the study. Twenty-nine young people aged 16–21 who had been trafficked into the UK from other countries completed the health survey. Participants originated from twelve countries, including Nigeria, Albania, and Slovakia. The length of time that had elapsed since they exited that situation ranged from three weeks to six and a half years. The median length of time since leaving the trafficking situation was 12 months (IQR 3–24 months), so most were commenting on experiences that were relatively recent. Table 1 shows that five of the participants were male and all but one of the young men were in the 20–21 age group, while the young women were more evenly spread across the 16–21 age range. The age at which young people were trafficked was calculated from questions that asked how long they had been in the trafficking situation and how long they had been out of it. Over half the group (17 of 29) were under 16, at the time they were trafficked, with the youngest age at which anyone was trafficked being six, and the oldest 20. The majority originated from African countries, with two-fifths (10) of the young women from Nigeria. Nearly a third (7) of the young women were Eastern European.

Although eight young people (7 female, 1 male) had been married or promised in marriage (including two young women who had been trafficked for marriage), only one described herself as currently married. Three young women had children who were living with them in the UK at the time of the interview.

Table 2 shows that three young people had no formal schooling, but most had continued their education into their teens. When asked about learning disabilities or difficulties reading in their own language, a third (8) of the young women reported a disability or reading difficulties. Eleven of the young women and all but one of the young men (4) described being hit, kicked or physically hurt prior to being trafficked. Eight of the young women said that they had been forced to have sex before they had been trafficked. Identified perpetrators included family member (1), recruiter (1), acquaintance (1) and stranger (1). Despite the fact that most had received schooling in their teens, this group of young people appears to have had some key vulnerabilities which may have exposed them to trafficking.

### 3.2 Experiences of trafficking

The length of time the young people had remained within the trafficking situation ranged from two weeks to eight years. The median duration of exploitation was 12 months (IQR 4, 42). Although some young people experienced more than one type of exploitation, the main type of exploitation experienced while trafficked is shown in Table 3.

Table 3 shows that sex work was by far the largest category of work into which young people were trafficked followed by domestic servitude (e.g. child care or housekeeping). It is worth noting that two young men were exploited in sex work and domestic servitude. This reflects the gender balance in this group of respondents but it also highlights that, for the majority of this group of vulnerable young people, sexual exploitation and its consequences were central experiences. Sex work and domestic service are, of course, frequently characterised by informal and illegal working arrangements and are particularly resistant to inspection or regulation. Only two of the young people reported working eight hours or less a day. Thirteen said that they had no fixed hours. Only four had one or more rest days a week when working.

This group had experienced high rates of violence and threats whilst trafficked (see Table 4). Twenty-four (21 female; 3 male) reported being physically hurt and 16 young women had received an injury. Eighteen young people, including all but two of the young women and two young men, had been forced to have sex. Sexual violence was experienced by both young women and by young men and by those trafficked for forced sex work and those exploited as domestic workers and in other labour sectors. Threats were reported by 26 of the 29 young people (22 female; 4 male) who stated they were physically threatened and eleven were threatened with harm to their family. Two-thirds (17 female; 3 male) said they were still scared of their traffickers.

They also reported considerable restrictions of liberty and deprivation, as shown in Table 5. Fifteen young women and three young men had been confined in a locked room while seventeen had been denied access to their passport or identity documents. Thirteen (11 female; 2 male) had nowhere to sleep or had slept on the floor and 12 (10 female; 2 male) described sleeping in overcrowded conditions. Nearly half of the participants (14) said that they had had no clean clothing and six described lacking basic hygiene facilities. Eleven said they did not have sufficient food while four reported insufficient water.

A small number described being forced to drink alcohol (8) or take drugs (4), and three were forced to take medication while trafficked.

### 3.3 Health and mental health

#### 3.3.1 Pregnancy and sexual health

Pregnancy and sexual health conditions represented life-changing issues for the young women, and these were experienced by those trafficked into other settings as well as those trafficked for sexual exploitation. Five young women had become pregnant while trafficked: three of these were working in the sex industry; one was trafficked into domestic servitude, and one for labour exploitation. Two had an abortion, two were currently pregnant and one had given birth and had her child with her. None had seen a midwife whilst they were in the trafficking situation. A further two young women had had children since leaving the trafficking situation. Four young women reported having been previously diagnosed with sexually transmitted infections and two had been diagnosed with HIV. This last group included young women trafficked for sexual exploitation, domestic servitude and labour exploitation.

#### 3.3.2 Physical health

Table 6 shows that over half the young people (15 female, 1 male) described being bothered by headaches in the last four weeks and nine (8 female, 1 male) reported memory problems. Seven (6 female, 1 male) had been worried by stomach pains and seven young women noted back pain, including one of the three young women who were pregnant at the time of interview. Six young women had experienced dental pain.

#### 3.3.3 Mental health

Table 6 indicates that mental health disorders were found to be at a high level in this group of young people. Two-thirds (16 female, 3 male) screened positive for probable disorders, including PTSD. Over half the group (15 female, 1 male) had PTSD symptoms. The qualitative data collected after the survey was completed included accounts of feeling overwhelmed by memories of abusive experiences:

… if there was anything I could do just to clear the memory, I would do it. Just erase everything…all of it. (Young woman trafficked for domestic servitude, aged 19).

Twelve (11 female, 1 male) reported suicidal thinking in the last week. Two young people described recently attempting suicide, one whilst in detention. Both described difficulties in coping with the overwhelming feelings they were experiencing:

I tried suicide….Some people can’t open up and talk and then [I saw] a doctor who is a professional … you can talk and you can open up. (Young man trafficked for labour exploitation, aged 21).

When we examined the relationship between probable mental disorder and type of exploitation, we found that 15 of the 18 who had been sexually exploited, all those who had been trafficked into domestic servitude (7), and two of the three trafficked into labour exploitation scored positive for a probable mental disorder. There was considerable overlap between prior vulnerability and mental disorder with five of the eight who had been forced to have sex prior to being trafficked reporting symptoms indicative of a mental disorder, and seven of the eight who had difficulty with reading in their own language or a learning difficulty also reported symptom levels associated with a disorder. The small numbers involved here means it is inappropriate to measure *p* values but these findings suggest avenues for future research.

### 3.4 Young people’s experiences of barriers to accessing health services

The young people interviewed described a range of barriers to utilising health services. While they were in the trafficking situation, their access to health professionals and freedom to make decisions about healthcare was frequently restricted by traffickers. One young woman who had an abortion arranged by her traffickers said that she was encouraged by a nurse to approach the authorities and explain her situation but she feared for her safety if she followed this advice. Another young woman trafficked for sex work described how she was too frightened to explain her situation honestly to a sexual health worker:

They ask me why are you doing this, do you like doing this? I say ‘yes’ because I was scared. (Young woman trafficked for sex work, aged 17).

However, even when they had escaped from their traffickers, complex gatekeeping systems seemed to impede or delay their access to and use of health services; registration with general practitioners or family doctors (GPs) was described as particularly difficult:

It wasn’t easy, because my friend tried many times before to register me with GP because it was kind of an emergency. I needed to see a doctor because I was pregnant, but the GP wouldn’t register me without any papers from the Home Office, so we had to wait until that paper arrived and then I was registered. (Young woman trafficked for sex work, aged 21).

Language barriers appeared to exacerbate the challenges of dealing with complex and unfamiliar systems and organisations and the absence or limited availability of interpreters or reliance on telephone interpreting systems could make for difficulties in communicating directly with health professionals. Some young people expressed a preference for face-to-face interpreting services; for example, a young man who had experience of a telephone interpreting service remarked that “maybe it would be better if the interpreter came in person” (Young man trafficked for labour exploitation, aged 21).

Some young people interviewed reported feeling as if they were not listened to or believed or taken seriously by health professionals. As noted by one young woman who had to make repeated visits to her family doctor:

It’s good to listen to children …Check then what they say…when I had the, the pain in my throat, he [family doctor] didn’t give me medicine. (Young woman trafficked for sex work, aged 18).

### 3.5 Young people’s experiences of responsive health services

A few young people emphasised the importance of being able to make choices about their healthcare, including being able to request a female health practitioner. Some young women had found staff working in maternity services to be particularly helpful, and appreciated staff continuity and the opportunities this offered for developing trusting relationships. This young woman was very positive about her first experience of UK healthcare; she was admitted to hospital as an emergency very late in her pregnancy and found midwives attentive and reassuring:

Because they was there with me when I need them. (Young woman trafficked for sex work, aged 20)

Support workers from relevant organisations, foster carers and others, including friends, were described as playing a key role in advocating for young people’s health needs and assisting them to navigate services. One young woman described how her support worker would break down the health professional’s communication into comprehensible messages for her: she “put it in pieces for me so I will understand.” (Young woman trafficked for domestic servitude, aged 17).

Young people, who could remain fearful of traffickers even after their escape, wanted reassurances about confidentiality in their contacts with health services. They wanted to be given time to explain their needs and for their accounts to be respected:

The most important thing is to ask, and to give you time to explain how you are feeling instead of just assuming what is wrong, giving you the chance to explain, and listening to your opinion about why you feel like that. (Young woman trafficked for sex work, aged 17).

Whilst some, like the young woman quoted above, wanted to talk about their experiences, for others, talking evoked too many distressing thoughts. One young person with mental health problems explained that she did not want to discuss her past experiences of trafficking with her psychiatrist, but instead wanted to look ahead:

I want to forget what happened. I just want to move on. I just want to get my own flat and live and maybe get a job. (Young woman trafficked for domestic servitude, aged 19).

Another young woman’s desire to forget appeared to be linked to having been asked to repeat her story on numerous occasions to different people:

I’ve said my, everything I know, to police, to social worker, to social services, so… I’ve gone through a lot of things, so I don’t think I can say anything more, much like that anymore. I need to forget… I don’t want to remember them anymore. (Young Woman trafficked for sex work, aged 18).

For some young people, mental health or counselling services may need to be made available at a later date when they feel they need them or are more ready to participate. This requires repeated offers of such support, even if it is initially declined.

### 3.6 Professionals’ perspectives

Seven of the 52 professionals interviewed had experience of providing services for children and young people. Two were health professionals; two worked in specialist sexual health services and two worked in non-governmental organisations (NGOs) that offered advice and support services to trafficked children and young people. Six were female and one was male. Some key themes identified in these interviews reiterated and reinforced findings from the health survey. In discussing the challenges involved in identifying the health needs of trafficked people, interviewees noted that a substantial proportion of trafficked young people were themselves parents and that this was often a consequence of their exploitation:

… I probably see about a third of people that actually have children from their exploitation. And …a couple of them actually had children in the exploitation [setting]. They didn’t actually have the child in hospital. So that’s caused some health problems. (NGO Practitioner 1).

A health practitioner had encountered a number of large families where parents had been trafficked into the country and had brought their children with them:

The example of a whole family who’s been trafficked and actually the father is the one who’s been trafficked to work, with the promise of a better life and better job for his family…he brings the whole family with him and…they all end up in some way being trafficked and exploited. (Health Professional 1).

Interviewees agreed that sensitivity, attention to confidentiality and continuity of staff were needed from health professionals when engaging with trafficked children and young people:

…if I need them to go to a GUM [genitourinary medicine] clinic … I would always ring up and arrange a special appointment for them, so they’re seen by a senior doctor, they’re not sitting in with everybody else waiting in the waiting room. (Health Professional 2).

The best outcome is that they, you know have access to a GP and that they get to see the same person every time they go, and that a relationship is built up between them… if there has been sexual abuse… That might be something that they might talk to a doctor about, if they had a chance to kind of create a bit of familiarity and a bit of trust. (NGO Practitioner 2).

However, NGO practitioners did not consider that health professionals consistently provided a sensitive and responsive service. One interviewee noted that maternity staff had failed to make use of interpreters when working with a young woman who spoke no English. The other reported that health practitioners rarely made direct referrals to relevant specialist support services but rather relied on children’s social work services to access those services for children and young people. This could result in lengthy and convoluted referral routes whereby young people entered out-of-home care and were then registered with a GP who was expected to refer them to specialist services. This NGO practitioner argued that from the perspective of healthcare practitioners: “They’re seen first and foremost as an immigrant, and then as a young person with potential health needs.” (NGO practitioner 2).

The professionals interviewed concurred with the data showing high levels and prevalence of mental health need among trafficked children and young people. While they attributed much of this to the trauma and violence experienced when trafficked, they noted that post-trafficking isolation, poor living conditions and lack of support could also contribute to the development of mental health disorders. The age assessments undertaken by social workers to ascertain if young people were under 18 and entitled to health, education and social work services were identified as a particular source of stress and were perceived to contribute to the erosion of trust of statutory services:

It causes them a lot of stress…and also this, sort of, opinion of not [being] believed. Some talk about that…no-one believes them… It causes impact on them feeling valued, and it knocks their confidence a lot…they do often talk about having to tell their story again and again and again and no-one believing them and they’ve said the same thing lots of times and why aren’t people believing them. (NGO practitioner 1).

Practitioners agreed that, while some trafficked young people wanted and were keen to use mental health or counselling services, others needed to ‘move on’, whilst others needed to wait until they were ready to use this type of support. However, they were often not offered repeat opportunities to access these services:

I think a lot of the time a child will say, or a young person will say, ‘I don’t want counselling now, it’s not for me,’ and then it’s like, ‘Oh, well they’ve refused counselling,’ and it will never be readdressed. (NGO practitioner 1).

Both the NGO practitioners and health professionals noted the ‘huge gap’ in availability of mental health services for this group and described long waiting lists with services being “completely oversubscribed: throughout the country, there are not enough mental health services.” (Health Professional 2).

## 4 Discussion

The majority of young people participating in this study had experienced serious physical harm or threats whilst trafficked as well as some form of restriction of liberty and/or deprivation. In addition, sexual violence was a widespread experience among this group and this was not confined to those working in the sex industry, but was also experienced by young women trafficked into domestic servitude and by young men. This indicates how important it is that health professionals assess for sexual violence and address the sexual health needs of all young trafficking survivors, regardless of gender or type of exploitation. The high level of mental health needs among the trafficked young people surveyed was striking but is consistent with the findings of Kiss et al.’s (2015) Mekong study. There was an indication that prior vulnerability in the form of learning difficulties or earlier experiences of sexual abuse might be related to vulnerability to being trafficked and a study of the health needs of women and adolescents trafficked into Europe (Zimmerman et al., 2003) highlights that pre-trafficking experiences of violence and abuse can make women a target for traffickers as well as possibly contributing to their motivation to leave home. A history of abuse may also increase susceptibility to physical illness and mental health problems.

Addressing young people’s mental health needs will be a priority for planning service provision. However, professionals interviewed expressed concerns about the availability of children’s mental health services in a climate where service thresholds are increasingly high, so that only those children and young people whose mental health needs have reached crisis point receive a service; this feature of the service landscape has been highlighted by other research (Franklin & Doyle, 2013). It was notable that three of the young women participating in this study were mothers; their mental health needs might have implications for their parenting. Reconceptualising trafficked people as parents provokes consideration of the impact of the trauma experienced on their parenting and on their children’s development. Offering appropriate health services and support to facilitate access to and take up of those services for trafficked young people may represent a means of intervening early in the lives of families whose histories are marked by exploitation and violence. Over half of the adults participating in the study of adult trafficked people included in the same research programme (see Oram et al., 2016) were found to be parents but little is as yet known about how trafficking-related trauma might impact on parenting and on the children of trafficked people.

Professionals’ attitudes also appeared to influence the delivery of healthcare to this group. Confidentiality and sensitivity from those delivering services are key and some groups of health practitioners, such as those working in sexual health services, may be more attuned to the need for such approaches than others. Austerity policies have resulted in increased restrictions on access to health and other public services for those from outside the UK (House of Lords and House of Commons, Joint Committee on Human Rights, 2015). A climate where health providers are being asked to function as gatekeepers to deny access or collect charges for healthcare from migrants can create confusion and uncertainty regarding rights to receive services for health staff dealing with trafficked young people (Westwood et al., 2016).

When services were accessed, some young people felt that health professionals did not take them seriously and practitioners interviewed suggested that trafficked young people’s experiences of age assessments undertaken by children’s social services contributed to their sense that they lack credibility. The damaging impact of these assessments on young people’s relationships with professionals has been documented by other studies (Chase, Knight, & Statham, 2008; Crawley and Kohli, 2013, Franklin and Doyle, 2013). Healthcare staff need relevant training to enable them to ask appropriate questions in a sensitive and respectful manner. As Pearce (2011) notes, trafficked young people have often undergone a rapid transition to adulthood as a consequence of enforced separation from family and experiences of war and trauma, they are used to making difficult decisions on their own and their capacity for making choices needs to be acknowledged. However, it is also important that services such as counselling or psychiatry which may not be appropriate or acceptable in the near aftermath of trauma are offered again when survivors are more prepared to engage in these types of support. Kohli and Mather’s (2003, p. 208) study of young refugees in the UK suggested that “young people want to face the present first, the future next and the past last.”

The young people interviewed had also encountered considerable barriers to accessing health services, and interviews with practitioners suggest that health professionals need to be better informed about and prepared to refer children and young people directly to specialist services. In the UK, children and young people who have been trafficked are defined as in need of child protection services and this means that health professionals will refer those under 18 directly to social workers. Whilst this procedure has resulted in clearer referral pathways for trafficked children and young people than are available for adults (Hemmings et al., 2016), it may also have contributed to a mindset whereby trafficked children and young people are considered ‘someone else’s business’. At present, some trafficked young people have to experience unnecessarily long and complex routes to specialist services.

Support workers from specialist voluntary organisations and others such as foster carers emerged as playing a crucial role in assisting young people to access health services. Clearly, health professionals need to ascertain the identity of such people to ensure that they are not traffickers but their contribution emerged as key in ensuring good communication between health professionals and anxious or fearful young people. Both young people themselves and professionals interviewed emphasised the value of support and advocacy for these young people being delivered in the context of a trusting relationship built over time with an identified individual. A scheme to provide legal guardians who could fulfil this role for trafficked children and young people has been successfully piloted in Scotland (Crawley & Kohli, 2013) and is, at the time of writing, being tested in England; it was implemented in Northern Ireland in 2015 (ECPAT, 2015). Most adolescents need guidance and assistance to access healthcare services and trafficked young people are particularly disadvantaged in this respect by their lack of familiarity with local healthcare systems, language barriers, frequent changes of address, high levels of trauma and mental health needs, ongoing fear of traffickers and confusion surrounding their legal status and entitlement to free healthcare in the UK. Crawley and Kohli’s (2013) evaluation of the Scottish Guardianship pilot includes accounts of Guardians assisting young people to understand the roles of different health providers, providing encouragement and support to access mental health services, accompanying them to specialist appointments and reinforcing and contributing to ongoing interventions.

Finally, while this study was able to provide important evidence on the health needs of child trafficking survivors, this mixed methods study has some limitations. We have no means of knowing how representative our sample of trafficked young people was and we were unsuccessful in attempts to recruit young people under 16. However, this is, to our knowledge, the largest study of trafficked young people in a high income country to investigate directly young people’s health experiences and perceptions of services. We have also been able to draw together different data sources to address the question of what might constitute an appropriate response from healthcare services.

## 5 Conclusion

This study found that young people who survive extreme forms of exploitation are often in need of urgent, as well as ongoing healthcare, especially mental health support. Yet there appear to be many barriers associated with accessing and using services, as well as challenges for the professionals providing care. At the time of writing, Europe is experiencing an influx of unaccompanied children on a scale that has not been seen since World War II and concerns about their vulnerability to trafficking were articulated in a debate held at ISPCAN’s 2015 European Regional Conference in Bucharest. There is substantial reason to believe that health services in the UK will be encountering increased numbers of highly exploited children among these unaccompanied minors. Health services should be prepared to meet the needs of these children and young people who, in escaping violence and oppression at home, may be trafficked for exploitation in the sex industries, private households, industries and agriculture of high income countries. Policy makers need to work towards finding better ways to help some of the most vulnerable young people to access the healthcare they clearly require. In addition to the very clear health arguments to be made in favour of doing the utmost to provide much-needed healthcare to children and young people, there are also humanitarian and moral arguments currently being voiced in the UK and across Europe.

The knowledge base in respect of the health needs of trafficked young people requires further development. Research is particularly needed to establish which interventions are effective to address children’s and young people’s various mental health needs, especially their longer term outcomes and what might contribute to their coping and resilience.

## Figures and Tables

**Table 1:** Age at interview and gender (n = 29).

Age at Interview	Female (n = 24)	Male (n = 5)	Total (n = 29)
16–17	6	1	7
18–19	11		11
20–21	7	4	11
Total	24	5	29

**Table 2:** School Leaving Ages of Participants (n = 29).

Age on leaving school	No of participants (n = 29)
No formal schooling	3
8–11	2
12–15	5
16–19	9
Currently attending	6
Missing	4
Total	29

**Table 3:** Main Type of Exploitation When Trafficked, by Gender (n = 29).

Type of exploitation	Males n = 5	Females n = 24	Total n = 29
Sex work	1	14	15
Forced marriage	0	3	3
Domestic servitude	1	6	7
Agricultural labour	0	1	1
Car washing	1	0	1
Factory labour	1	0	1

**Table 4:** Violence Experienced When Trafficked by Gender (n = 29).

Violence during trafficking	Males n = 5	Females n = 24	Total n = 29
Threats to self while trafficked	4	22	26
Threats to family while trafficked	1	10	11
Physical violence while trafficked	3	21	24
Sexual violence while trafficked	2	16	18
Injury while trafficked	0	16	16

**Table 5:** Experiences of Deprivation while Trafficked by Gender (n = 29).

Experiences during trafficking	Males n = 5	Females n = 24	Total n = 29
Confined in a locked room	3	15	18
Denied access to their passport or identity documents	1	16	17
Nowhere to sleep or had slept on the floor	2	11	13
Sleeping in overcrowded conditions	2	10	12
No clean clothing	2	12	14
Lacking basic hygiene facilities	1	5	6
Lacked sufficient food	2	9	11
Lacking sufficient water	1	3	4

**Table 6:** Experiences of Physical and Mental Health Problems Post-Trafficking (n = 29).

Physical and mental health	Males n = 5	Females n = 24	Total n = 29
Headaches in the last 4 weeks	1	15	16
Memory problems in the last 4 weeks	1	8	9
Back pain in the last 4 weeks	0	7	7
Stomach pain in the last 4 weeks	1	6	7
Dental pain in the last 4 weeks	0	6	6
Psychological distress^a^	3	16	19
PTSD	1	15	16
Suicidal thinking in last week	1	11	12

a 1 or more above threshold scores on the PHQ-9, GAD-7, or SDQ emotional difficulties, conduct difficulties, or hyperactivity subscales.

## References

[bib0005] CEOP (2007). A scoping project on child trafficking in the UK.

[bib0010] CEOP (2009). Strategic threat assessment: child trafficking in the UK.

[bib0015] Chase E., Knight A., Statham J. (2008). The emotional well-being and mental health of unaccompanied young people seeking asylum in the UK.

[bib0020] Cole J., Sprang G. (2015). Sex trafficking of minors in metropolitan, micropolitan, and rural communities. Child Abuse and Neglect.

[bib0025] Crawford M., Kaufman M.R. (2008). Sex trafficking in Nepal: Survivor characteristics and long-term outcomes. Violence Against Women.

[bib0030] Crawley H., Kohli R. (2013). ‘She endures with me’: an evaluation of the Scottish guardianship service pilot.

[bib0035] Crawley H. (2007). When is a child not a child? Asylum, age disputes and the process of age assessment.

[bib0040] Department for Children, Schools and Families (DCSF) (2007). Safeguarding children and young people who may have been trafficked.

[bib0045] ECPAT UK (2015). Northern Ireland pioneers legal guardianship for trafficked children. 20 January 2015. http://www.ecpat.org.uk/media/northern-ireland-pioneers-legal-guardianship-trafficked-children.

[bib0050] Franklin A., Doyle L. (2013). Still at risk: a review of support for trafficked children.

[bib0055] Hemmings, S., Jakobowitz, S., Abas, M., Bick, D., Howard, L. M., & Stanley, N., et al., Responding to the Health Needs of Survivors of Human Trafficking: a Systematic Review, 16, 2016, 320, doi: 10.1186/s12913-016-1538-8. 10.1186/s12913-016-1538-8 PMC4966814 27473258

[bib0060] Home Office (2014). Review of the National Referral Mechanism for victims of human trafficking. http://https://www.gov.uk/government/uploads/system/uploads/attachment_data/file/372960/Review_of_the_National_Referral_Mechanism_for_victims_of_human_trafficking.pdf.

[bib0065] House of Lords and House of Commons, Joint Committee on Human Rights (2015). The UK’s compliance with the UN convention on the rights of the child. Eighth report of session 2014–2015. HL Paper 144, HC 1016.

[bib0070] International Labour Office (ILO) (2012). Global estimate of forced labour: results and methodology.

[bib0075] Kiss L., Pocock N., Naisanguansri V., Soksreymom S., Dickson B., Thuy D. (2015). Health of men, women, and children in post-trafficking services in Cambodia, Thailand, and Vietnam: An observational cross-sectional study. Lancet Global Health.

[bib0080] Kohli R., Mather R. (2003). Promoting psychosocial well-being in unaccompanied asylum seeking young people in the United Kingdom. Child and Family Social Work.

[bib0085] Kroenke K., Spitzer R.L., Williams J.B. (2001). The PHQ-9: validity of a brief depression severity measure. Journal of General Internal Medicine.

[bib0090] Lewis G., Pelosi A., Araya R., Dunn G. (1992). Measuring psychiatric disorder in the community: The development of a standardised assessment for use by lay interviewers. Psychological Medicine.

[bib0095] Miller C.D., Campbell J.C. (1993). Reliability and validity of the Miller Abuse Physical Symptom and Injury Scale (MAPSAIS).

[bib0100] Munro E.R., Holmes L., Ward H. (2005). Researching vulnerable groups: Ethical issues and the effective conduct of research in local authorities. British Journal of Social Work.

[bib0105] National Crime Agency (NCA) (2015). National referral mechanism statistics—end of year summary 2014. http://www.nationalcrimeagency.gov.uk/publications/national-referral-mechanism-statistics/502-national-referral-mechanism-statistics-end-of-year-summary-2014/file.

[bib0110] National Centre for Social Research and University of Leicester (2011). Adult psychiatric morbidity survey, 2007.

[bib0115] The National Survey Of Sexual Attitudes and Lifestyles (Natsal) (2015). The national survey of sexual attitudes and lifestyles questionnaire, Revised for Natsal3. http://natsal.ac.uk/home.aspx.

[bib0120] Noy C. (2008). Sampling knowledge: The hermeneutics of snowball sampling in qualitative research. International Journal of Social Research Methodology.

[bib0125] Oram S., Khondoker M., Abas M., Broadbent M., Howard L.M. (2015). Characteristics of trafficked adults and children with severe mental illness: A historical cohort study. The Lancet Psychiatry.

[bib0130] Oram S., Abas M., Bick D., Boyle A., French R., Jakobowitz S. (2016). Human Trafficking and Health: A cross-sectional survey of male and female survivors in contact with services in England. American Journal of Public Health.

[bib0135] Ottisova L., Hemmings S., Howard L.M., Zimmerman C., Oram S. (2016). Prevalence and risk of violence and the mental, physical and sexual health problems associated with human trafficking: An updated systematic review. Epidemiology and Psychiatric Sciences.

[bib0140] Pearce J.J. (2011). Working with trafficked children and young people: Complexities in practice. British Journal of Social Work.

[bib0145] Prins A., Ouimette P., Kimerling R. (2004). The primary care PTSD screen (PC-PTSD): Development and operating characteristics. Primary Care Psychiatry.

[bib0150] Ross C., Dimitrova S., Howard L.M., Dewey M., Zimmerman C., Oram S. (2015). Human trafficking and health: A cross-sectional survey of NHS professionals’ contact with victims of human trafficking. BMJ Open.

[bib0155] Smith J., Firth J. (2011). Qualitative data analysis: The framework approach. Nurse Researcher.

[bib0160] Spitzer R.L., Kroenke K., Williams J.B.W., Lowe B. (2006). A brief measure for assessing generalized anxiety disorder. Archives of Internal Medicine.

[bib0165] United Nations (2000). United nations, protocol to prevent, suppress and punish trafficking in persons, especially women and children, supplementing the united nations convention against transnational organized crime.

[bib0170] Varma S., Gillespie S., McCracken C., Greenbaum V.J. (2015). Characteristics of child commercial sexual exploitation and sex trafficking victims presenting for medical care in the United States. Child Abuse and Neglect.

[bib0175] Westwood, J., Howard, L. M., Stanley, N., Zimmerman, C., Gerada, C., & Oram, S., (forthcoming). Access to and experiences of healthcare services by trafficked people: findings from a mixed methods study in England. *British Journal of General Practice*. 10.3399/bjgp16X687073 PMC5072917 27672141

[bib0180] Zimmerman C., Yun K., Shvab I., Watts C., Trappolin L., Treppete M. (2003). The health risks and consequences of trafficking in women and adolescents findings from a european study.

